# Childcare and depression during the coronavirus pandemic in South Africa: A gendered analysis

**DOI:** 10.1371/journal.pone.0255183

**Published:** 2021-08-06

**Authors:** Chijioke O. Nwosu

**Affiliations:** The Impact Centre, Human Sciences Research Council, Cape Town, Western Cape, South Africa; Shahjalal University of Science and Technology, BANGLADESH

## Abstract

**Background:**

The 2019 coronavirus disease (COVID-19) pandemic resulted in the closure of businesses and schools, the remote provision of services and the disruption of the services of professional childminders. These disruptions resulted in a significant increase in parental responsibility for childcare. Such a substantial increase in time requirements for childcare domestically has potential mental health consequences. We therefore ascertained the relationship between childcare and depression in South Africa during the pandemic.

**Methods:**

Data came from the National Income Dynamics Study-Coronavirus Rapid Mobile Survey, a longitudinal telephonic survey conducted during the COVID-19 pandemic in South Africa. The outcome was a depression index obtained from the two-item Patient Health Questionnaire while the main covariate was the average number of hours spent in taking care of children per weekday. We employed the ordered logit model.

**Findings:**

We found a positive relationship between spending more hours on childcare and worse depressive health for caregivers in both periods analyzed. Childcare responsibilities preventing/mitigating the ability of caregivers to work as well as preventing caregivers from searching for jobs moderated the depression-childcare relationship.

**Conclusion:**

These findings highlight the need to carefully consider policy responses aimed at containing the pandemic. We advocate a multi-stakeholder approach to mitigating the mental health impact of COVID-19 by encouraging more collaboration between government, school authorities, employers and parents/guardians.

## Introduction

The 2019 coronavirus disease (COVID-19) pandemic has had a devastating impact on livelihoods. While significant attention has rightly focused on the dangers of infection and the economic consequences of the pandemic due to job losses, etc., scholars have begun to highlight the nontrivial mental health effects of the pandemic. A focus on the mental health consequences of the pandemic is important for a number of reasons. One, the fear of infection as well as the endless news cycles highlighting the gloomy reality surrounding the pandemic can worsen mental health [1]. Second, some measures taken to combat the pandemic, like lockdowns, have serious short- and long-term mental health implications [2]. Moreover, the massive job losses occasioned by the pandemic resulted in a worsening of mental health outcomes [3, 4]. Furthermore, some direct measures taken against the pandemic, like school closures, resulted in secondary consequences like significantly increased time requirements for childcare, which can potentially have adverse mental health consequences.

South Africa implemented one of the most stringent response measures against the pandemic in Africa and globally [5]. This chiefly consisted of a declaration of a national state of disaster on 15 March 2020 and the implementation of a tiered series of nationwide lockdowns beginning with level 5 –the highest and strictest level–on March 26 [6]. Level 5 lockdown, which lasted until April 30, 2020, proscribed non-essential travel and gathering including for work. The restrictions were lowered to level four which entailed restrictions on most non-essential travel and gatherings between May 1 and May 31, 2020. Further lowering of the restrictions to levels 3, 2 and 1 (with level 1 being the least restrictive) were implemented during the periods, June 1-August 17, 2020, August 18-September 20 2020, and September 21-December 28 2020 respectively. Following a recent spike in cases, the government placed the country on an adjusted level 3 lockdown from December 29 2020 [7].

One direct consequence of the lockdowns is substantial job losses. One estimate indicates that about three million jobs were lost between February and April 2020 [8]. Unfortunately, most of these jobs had yet to return as at June 2020, implying prolonged agony for the affected workers and their families, with the lowest employment levels and the slowest recoveries experienced by disadvantaged groups like women and historically marginalized racial groups [9]. A significant number of those who remained in their jobs experienced reduced incomes, a phenomenon not unique to South Africa [10, 11]. Data from the third wave of the National Income Dynamics Study (NIDS)-Coronavirus Rapid Mobile (CRAM) survey (the dataset used for the analysis in this paper) indicate that even as of October 2020 (when the country was on level 1 lockdown restrictions), 74% of adults co-resident with children less than 7 years old indicated that neither them nor anybody in their household could afford Early Childhood Development (ECD) fees. For those who paid for ECD services in February 2020 (before the pandemic control measures were enacted), almost half (49%) could no longer afford them in October 2020.

School closures were an important part of the South African government and global response to COVID-19. Ewing and Vu [12] note that the pandemic was responsible for the suspension of schools in 189 countries as at April 2020, thereby placing a huge childcare strain on families. In South Africa, even among the schools where teaching and learning continued early on in the pandemic, such could only be conducted remotely. Similarly, the strict lockdown regulations meant that many daytime childminders discontinued work given that such work was not classified as essential. Anecdotal evidence also indicates that even before the strict lockdown regulations were enacted, some households asked for the temporary discontinuation of the services of daytime childminders for fear of infecting their households.

An obvious consequence of these school-related measures, the disruption of the work of childminders, job losses and reduction in hours supplied (resulting in household budget constraints) was a significant increase in the amount of time devoted by parents and guardians to childcare. As shown in wave 3 of the NIDS-CRAM survey, 70% of individuals co-resident with under-7 children indicated that children in their households did not attend ECD centres in the last seven days. For these children, COVID-19 and financial reasons accounted for at least 53% of the main reason for non-attendance. It is therefore, not unlikely that parents/guardians would need to increase the time they spent on children, be it in the form of play time, assisting with schoolwork (more than in the time of in-person learning), etc. Indeed, when asked who took care of the children who did not attend ECD centres, 92% indicated that it was either them or another adult in the household, with only 1.6% indicating that a domestic worker, nanny or childminder looked after such children.

Prior research indicates that this extra childcare burden disproportionately affected women in South Africa. According to Casale and Shepherd [13], who used the first two waves of NIDS-CRAM, this gendered burden of childcare was the result of women being more likely than men to live with children, while even among women and men living with children, the former reported spending more additional time on childcare especially during the early days of the lockdown than the latter. Even with the phased re-opening of schools due to the relaxation of lockdown restrictions and the subsequent reduction in childcare hours for both men and women, men experienced a sharper reduction in childcare responsibilities than women between April and June 2020. Among women and men living with children, the gender gap in childcare rose between April and June 2020 to the disadvantage of women [13]. (As will be seen in the analysis below, these childcare-related gender differences were either not as pronounced or largely reversed between the second and third waves of the NIDS-CRAM survey). Furthermore, the same study reported a significant gendered labour market impact of such extra childcare with more than twice the number of women as men reporting that childcare prevented them from working or made working difficult in June 2020.

Such higher female housework/childcare burden is not surprising. Though cultural norms regarding gendered household division of labour may be shifting, available literature clearly indicates a pervasive cultural context where the bulk of housework is performed by women in South Africa. For instance, a 2019 Oxfam report indicates that among 30-year-old South Africans, while men spent about 1.8 hours per day doing housework, women spent almost 5 hours [14]. Such a substantial gender gap in unpaid housework is no doubt driven at least in part by a set of norms where men are seen as breadwinners and women as responsible for taking care of the home, a belief system that has been significantly challenged by the reality of double earner households that is currently gaining ground in the country [15].

Obviously, such added responsibility has the potential to exert a substantial mental health toll on parents/guardians. Such mental health impacts are not only unique to South Africa. For instance in Poland, a survey of parents on the home education of their children showed that a significant number felt that home schooling was beyond their capabilities and were not confident in their ability to effectively home-school their children, with many expressing anxiety about their children’s future [16].

Zhao, Guo [17] evaluated the effects of home schooling due to COVID-19 on school children, parents and teachers in China. The study found that 17.6% of the students were suspected to have emotional or behavioural problems, while parents and teachers showed higher-than-usual levels of anxiety.

In the UK, Chandola, Kumari [18] reported a 30% increase in the prevalence of common mental disorders (CMD) between 2017/19 and April 2020, with the incidence of CMD dropping to below 13% from April to May 2020. They ascribed some of the increase in CMD between April and May to increased childcare and home-schooling demands.

While we are not aware of studies that have analysed the relationship between childcare and depression during the COVID-19 pandemic in South Africa, some studies have found a significant rise in the prevalence of adverse mental health during the pandemic relative to the pre-pandemic era. Oyenubi and Kollamparambil [19] found that the prevalence of depressive symptoms in South Africa doubled between 2017 and June 2020. Some of the key determinants of higher depressive symptoms were employment status and risk perception of contracting COVID-19.

How is (increased) childcare responsibilities and housework in general related to parental/caregiver’s mental health? One point well demonstrated in the literature is that housework in general often appears invisible and undervalued, hence one of the reasons that women, who are more likely to engage in such tasks, are more likely to report psychological distress than men [20]. The undervalued nature of the task coupled with the associated stress no doubt lead to heightened mental health disorders. Thus, it is not surprising that even among women, those with young children have been found to have worse mental health outcomes [21]. And when people are forced to combine housework/childcare with paid employment, there is the possibility of role overload, which can lead to poorer mental health outcomes [20]. This is especially true during the present pandemic where parents and caregivers report heightened anxiety due to additional childcare responsibilities [16].

Given the foregoing, this study ascertained the relationship between time spent on childcare and depressive symptoms during the COVID-19 pandemic in South Africa. We hypothesized that spending more time on childcare responsibilities was associated with worse depressive symptoms. Given the aforementioned gendered aspect of childcare responsibilities, we conducted a gendered analysis to determine if the relationship was stronger among men or women. Furthermore, given the above fact that such extra childcare responsibilities adversely affected participation in the labour market, we tested possible labour market avenues through which childcare might have been associated with mental health. Specifically, we tested whether it occurred through forcing caregivers to quit their jobs/made work difficult, reducing hours of work or preventing job seekers from searching for employment. Our results indicate that the labour market link operated through forcing people (especially men) to quit their jobs/made work difficult and preventing caregivers from searching for jobs.

This study is significant in a number of ways. First, it provides further evidence of the health impact of COVID-19. This is important given that much of the debate around the closure of schools has focused on its effect on children’s educational attainment and exposure to the virus. While these are hugely important, it is equally important to consider the wider mental health consequences of school closures which may have both short- and long-term consequences on the welfare of parents/guardians. Moreover, it further highlights the pandemic-mental health-labour market nexus which is very important when thinking through important policies like school closures and flexible work arrangements. Finally, this paper is an important resource as the world in general and South Africa in particular enter a second wave of the pandemic. It is important to find a healthy balance between pandemic control and the mental health of parents and caregivers who will bear the brunt of some of these public health measures.

## Materials and methods

### Data and key variables

Data came from the NIDS-CRAM survey, a rapid telephonic longitudinal survey of South Africans conducted during the COVID-19 pandemic. It was based on the last adult wave of the NIDS survey, the first nationally representative longitudinal survey of South Africans. (NIDS was conducted roughly biennially from 2008, with the fifth and final wave conducted in 2017. A top-up sample was included in wave 5 of the survey due to non-random attrition [22]).

The first wave of NIDS-CRAM targeted about 17,000 individuals while the sampling methodology was stratified sampling with batch sampling. Sampled individuals were sent to fieldwork teams in batches of 2,500 individuals randomly drawn from 99 strata. Using batch sampling allowed for flexibility given that it allowed for the adjustment of the sampling rate for each stratum with new information over the course of the survey [23]. The first wave of NIDS-CRAM, which was conducted in May-June 2020, successfully interviewed 7,073 respondents. Currently, NIDS-CRAM has three waves, with the second and third waves conducted in July-August and November-December 2020 respectively [24–26].

This study was based on the second and third waves of the NIDS-CRAM panel given that mental health was only elicited in those waves [27, 28]. Out of the 7,073 successfully completed interviews in wave 1, wave 2 recorded 5,676 successful interviews (80.2% success rate) while wave 3 recorded 5,046 successful interviews from the original wave 1 sample (representing 88.9% of successful interviews in wave 2 and 71.3% of the original successful interviews in wave 1) [24, 25]. (Due to attrition between NIDS-CRAM waves 1 and 2, wave 3 of NIDS-CRAM includes a top-up sample randomly drawn from the original wave 5 sample of NIDS [25]).

The outcome variable is a measure of depressive health derived from the two-item version of the Patient Health Questionnaire (PHQ-2) instrument. Respondents were asked the following questions: “Over the last two weeks, have you had little interest or pleasure in doing things?”; and “Over the last two weeks, have you been feeling down, depressed or hopeless?”. Responses were as follows: Not at all (0), Several days (1), More than half the days (2), and Nearly every day (3). These responses were codified to yield an index ranging from 0 to 6, with higher values indicating poorer mental health. While some authors suggest that a cut-off of 2 or 3 indicates the presence of possible depressive symptoms [29, 30], we follow Zuvekas [31] in using the linear PHQ-2 index given that it measures both probable clinical depression and depression severity.

The main covariate is the number of hours devoted to childcare on a typical weekday. The reference period for wave 2 was June 2020, which coincided with level 3 lockdown restrictions. For wave 3, the reference was October 2020, i.e. during level 1 lockdown restrictions. Other covariates were gender, race, education, location, an indicator for whether income decreased over the past four weeks, household’s experience of hunger, the type of dwelling the household lived in, respondents’ perception of their risk of COVID-19, marital status, age, household receipt of government grant(s), and the number of children in the household. The estimation samples consisted of 4,122 (4,331) observations in wave 2 (wave 3).

### Analytical methods

Given the ordered nature of the outcome variable, we estimated ordered logit regressions of the PHQ-2 index on hours spent on childcare and other covariates (see Table 2). The following generic model captures the relationship:

$$y_{i}=X^{/}\beta +\varepsilon_{i}$$

where y denotes the PHQ-2 depression score; *i* is an individual identifier; X is a vector of covariates; while *ε* denotes the idiosyncratic error term. We complement these models with insights from descriptive analyses.

## Results

### Descriptive statistics

**Fig 1:**
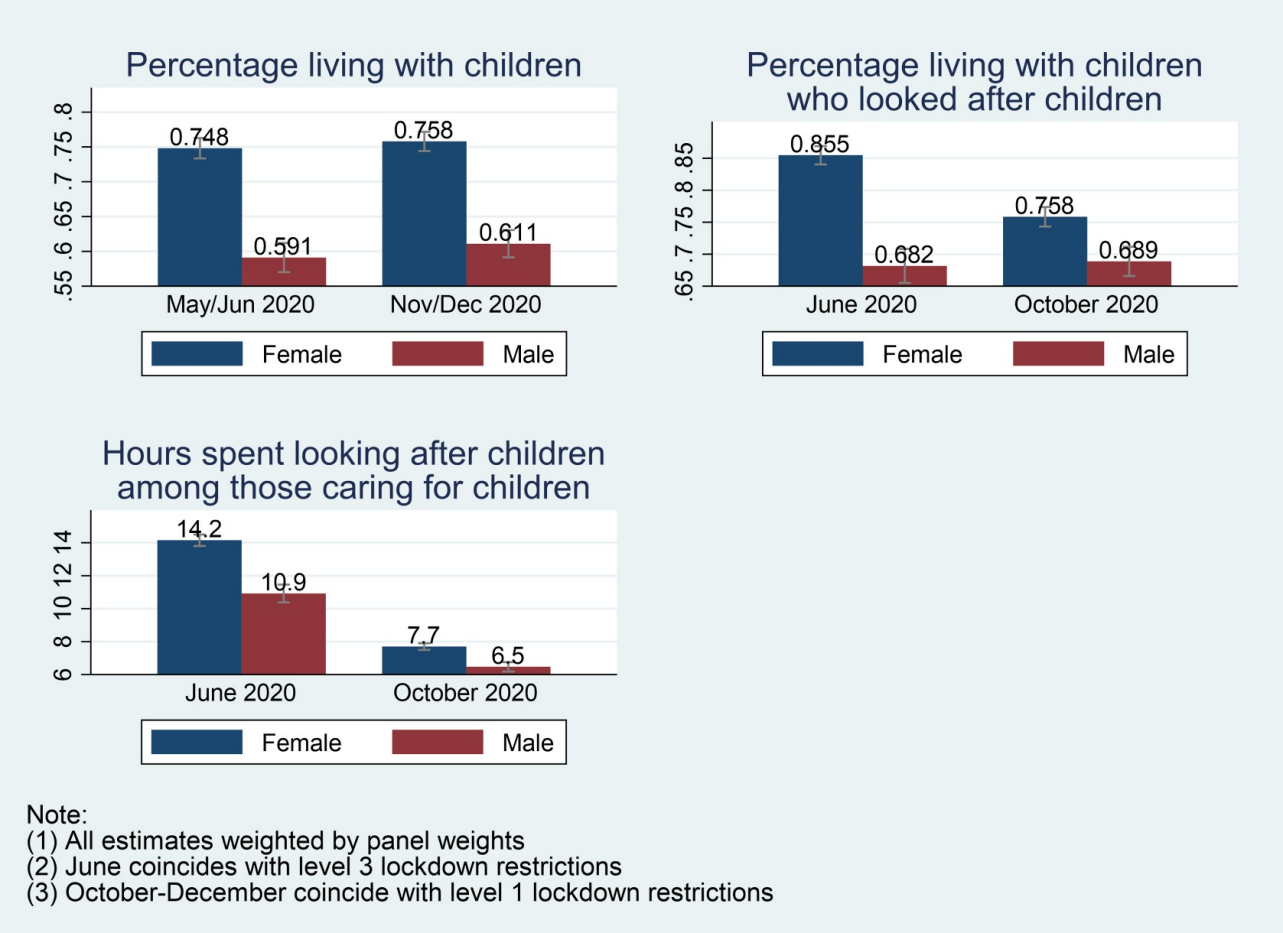
Depicts the gendered division of labour regarding childcare.

The percentage of women and men who lived in households that had children (aged 0–17 years) remained fairly constant, with 75% (76%) of women and 59% (61%) of men living in such households in wave 2 (wave 3). However, among these women and men, the percentage of women engaged in childcare reduced substantially compared to men. While the proportion of women engaged in childcare declined from 86% to 76% (a ten-percentage point drop), the proportion of men remained almost stagnant at 68% and 69% in wave 2 and wave 3 respectively. Not only did women record a relatively sharp decline in the proportion looking after children than men, women who looked after children also recorded a larger drop in their time commitment to those children than men between both waves. As shown in Fig 1, the average number of hours devoted to childcare per weekday by women who looked after children declined by 45.8% (from 14.2 hours to 7.7 hours) while it declined from 10.9 hours to 6.5 hours (a 40.4% decline) for men. However, women still spent significantly more time on childcare than men in both waves.

Table 1 presents the descriptive statistics indicating the gender-specific means/percentages and their gendered differences in each wave as well as the temporal differences in the statistics.

**Table 1:** Descriptive statistics.

	Wave 2				Wave 3				
	Female	Male	Diff (Fem-Male)	All	Female	Male	Diff (Fem-Male)	All	W3-W2
Variable	Mean/%	Mean/%		Mean/%	Mean%	Mean/%		Mean/%	
PHQ-2 depression score	1.3	1.3	-0.0	1.3	1.5	1.6	-0.0	1.6	0.2***
Childcare hours per weekday	8.9	4.3	4.6***	6.8	4.6	3.0	1.6***	3.8	-2.9***
Years of schooling	11.4	11.5	-0.1	11.5	11.3	11.4	-0.1	11.4	-0.1
Age	40.4	39.8	0.6	40.2	41.3	40.2	1.1	40.8	0.6**
Number of co-resident children	1.9	1.3	0.6***	1.7	2.1	1.5	0.6***	1.8	0.2***
Lives in formal location	30.4%	33.4%	-3.0	31.7%	30.1%	31.1%	-1.0	30.6%	-1.2
Lives in informal location	39.4%	42.1%	-2.8	40.6%	40.1%	43.5%	-3.4	41.7%	1.1
Lives in traditional location	17.2%	15.3%	1.9	16.3%	19.4%	18.1%	1.3	18.8%	2.4***
Lives on a farm or smallholding	13.0%	9.2%	3.8***	11.3%	10.4%	7.3%	3.1***	8.9%	-2.3***
Married or cohabiting	45.5%	56.4%	-10.9***	50.5%	45.1%	56.2%	-11.2***	50.3%	-0.2
Not employed	60.7%	42.4%	18.4***	52.4%	54.8%	36.8%	17.9***	46.4%	-5.9***
Household experienced a decrease in main source of income in past 4 weeks	16.8%	22.5%	-5.7***	19.4%	40.9%	29.7%	11.1***	35.7%	16.3***
Someone in household experienced hunger in last 7 days due to lack of food	16.0%	14.5%	1.5	15.3%	17.9%	15.5%	2.4	16.8%	1.5*
Lives in a house/flat (otherwise, traditional/ informal/ other type of house)	77.4%	81.3%	-3.9**	79.2%	78.4%	81.1%	-2.7	79.6%	0.5
Self-perceived no risk of COVID-19	42.8%	40.8%	2.0	41.9%	51.7%	50.9%	0.8	51.3%	9.5***
Self-perceived uncertain risk of COVID-19	13.2%	15.0%	-1.8	14.0%	10.6%	10.0%	0.6	10.3%	-3.7***
Self-perceived at risk of COVID-19	44.0%	44.2%	-0.2	44.1%	37.7%	39.1%	-1.4	38.3%	-5.8***
African	77.6%	75.6%	2.0	76.7%	76.8%	77.1%	-0.3	76.9%	0.2
Coloured	8.8%	10.6%	-1.8	9.6%	11.1%	10.2%	0.9	10.7%	1.1
Asian/Indian	1.9%	3.7%	-1.8	2.7%	2.0%	3.0%	-1.1	2.5%	-0.2
White	11.7%	10.2%	1.6	11.0%	10.1%	9.7%	0.5	9.9%	-1.1
Household member(s) received grant	74.9%	54.3%	20.6***	65.5%	76.3%	61.8%	14.5***	69.5%	4.0***
Male (otherwise, female)				45.6%				46.6%	1.0
**Number of observations**	**2,600**	**1,522**		**4,122**	**2,711**	**1,620**		**4,331**	

Note: All statistics weighted by wave 2 and wave 3 panel weights

*. **. *** indicate statistical significance at 10%. 5% and 1% level of significance.

Table 1 indicates no statistically significant difference in the PHQ-2 depression score between women and men in each wave. However, the scores significantly worsened over time. Women spent significantly more time caring for children in each wave than men; however, the gender differences declined significantly between wave 2 and wave 3 –as expected, the average times spent on childcare are lower in Table 2 than Fig 1 since the latter were conditional on spending time on childcare. Also, as expected with the re-opening of schools, the progressive lowering of lockdown restrictions and more people working again, there was a significant reduction in time devoted to childcare across both waves. Women lived in households with slightly (but significantly) more children than men. While men were more likely to live in households where the main source of income declined in wave 2, the converse obtained in wave 3. Regarding perception of personal risk of contracting COVID-19, while there was no gendered difference in each wave, there was a significant increase in the proportion of those who felt that they were not at risk of contracting the virus between wave 2 and wave 3. However, there were significant declines in the proportions of those who were either not sure of their risk of contracting the virus or felt they were at risk.

**Table 2:** Relationship between depression and childcare.

	(1)	(2)	(3)	(4)	(5)	(6)
	Wave 2 (level 3)			Wave 3 (level 1)		
Covariates	Female	Male	All	Female	Male	All
Childcare hours per weekday	0.018**	0.024**	0.019***	0.013	0.034*	0.023**
	(0.007)	(0.011)	(0.006)	(0.011)	(0.017)	(0.009)
**Location (Reference = lives in formal location)**						
Lives in informal location	0.046	0.051	0.038	0.297*	0.361*	0.331**
	(0.176)	(0.192)	(0.125)	(0.167)	(0.190)	(0.135)
Lives in traditional location	-0.202	0.339	0.000	0.065	0.017	0.055
	(0.183)	(0.251)	(0.145)	(0.183)	(0.225)	(0.139)
Lives on a farm or smallholding	-0.284	0.187	-0.090	-0.115	-0.136	-0.110
	(0.187)	(0.310)	(0.170)	(0.208)	(0.295)	(0.164)
Married or cohabiting	-0.070	-0.131	-0.140	-0.138	-0.176	-0.149
	(0.119)	(0.156)	(0.102)	(0.130)	(0.187)	(0.107)
Not employed	0.090	0.320*	0.163	-0.232*	0.022	-0.121
	(0.132)	(0.170)	(0.104)	(0.133)	(0.156)	(0.097)
Household experienced a decrease in main source of income in past 4 weeks	0.192	0.307	0.224	0.146	0.344**	0.242***
	(0.161)	(0.225)	(0.139)	(0.129)	(0.167)	(0.086)
Someone in household experienced hunger in last 7 days due to lack of food	0.823***	0.505**	0.670***	0.712***	0.856***	0.761***
	(0.145)	(0.227)	(0.131)	(0.118)	(0.154)	(0.095)
Lives in a house/flat (otherwise, traditional/ informal/ other type of house)	0.093	0.302	0.179	-0.063	-0.314	-0.184
	(0.159)	(0.201)	(0.137)	(0.206)	(0.200)	(0.164)
**Perception of COVID-19 risk (Reference = not at risk of contracting COVID-19)**						
Self-perceived uncertain risk of COVID-19	0.156	0.510**	0.303**	0.068	-0.529**	-0.204
	(0.163)	(0.246)	(0.141)	(0.196)	(0.232)	(0.154)
Self-perceived at risk of COVID-19	0.268*	0.288*	0.290**	0.406***	0.108	0.274***
	(0.152)	(0.158)	(0.118)	(0.125)	(0.167)	(0.104)
Years of schooling	0.038**	0.025	0.030**	0.022	-0.007	0.010
	(0.019)	(0.023)	(0.014)	(0.018)	(0.023)	(0.014)
Age (years)	-0.002	-0.019***	-0.008**	0.002	-0.001	0.001
	(0.004)	(0.006)	(0.004)	(0.005)	(0.007)	(0.004)
**Race (Reference = African)**						
Coloured	0.976***	1.078***	1.014***	0.826***	1.271***	1.015***
	(0.275)	(0.366)	(0.180)	(0.181)	(0.293)	(0.186)
Asian/Indian	0.620	0.099	0.230	0.327	0.475	0.410
	(0.694)	(0.467)	(0.284)	(0.681)	(0.421)	(0.369)
White	1.050***	1.594***	1.253***	0.646*	1.236***	0.905***
	(0.276)	(0.364)	(0.217)	(0.348)	(0.325)	(0.240)
Male (otherwise, female)			0.108			0.108
			(0.116)			(0.093)
Household member(s) received grant	0.017	-0.158	-0.038	0.195	-0.002	0.086
	(0.181)	(0.185)	(0.123)	(0.177)	(0.173)	(0.121)
Number of co-resident children	-0.047	0.039	-0.009	0.025	0.048	0.033
	(0.034)	(0.048)	(0.027)	(0.033)	(0.039)	(0.027)
Cutoff 1	0.879**	0.528	0.779**	0.588	-0.013	0.373
	(0.442)	(0.454)	(0.321)	(0.470)	(0.487)	(0.344)
Cutoff 2	1.536***	1.120**	1.402***	1.261***	0.714	1.066***
	(0.453)	(0.450)	(0.323)	(0.472)	(0.493)	(0.349)
Cutoff 3	2.252***	1.825***	2.106***	2.033***	1.329***	1.761***
	(0.458)	(0.459)	(0.324)	(0.468)	(0.505)	(0.352)
Cutoff 4	3.213***	2.934***	3.126***	2.897***	2.349***	2.690***
	(0.483)	(0.469)	(0.328)	(0.475)	(0.508)	(0.351)
Cutoff 5	4.082***	3.769***	3.974***	3.884***	3.053***	3.533***
	(0.511)	(0.511)	(0.364)	(0.473)	(0.511)	(0.351)
Cutoff 6	4.520***	4.116***	4.369***	4.390***	3.420***	3.960***
	(0.528)	(0.547)	(0.386)	(0.467)	(0.520)	(0.349)
F	5.7	4.8	7.4	8.0	5.2	10.5
p	0	0	0	0	0	0
**Number of observations**	**2,600**	**1,522**	**4,122**	**2,711**	**1,620**	**4,331**

Note: Model is ordered logit; Outcome is PHQ-2 depression scores; All statistics account for survey design and non-random attrition using appropriate weights

*, **, *** indicate statistical significance at 10%, 5% and 1% levels of significance respectively; Standard errors in parentheses.

We present wave-specific regression results in Table 2. The results indicate a positive and significant association between the number of hours spent on childcare per weekday and the PHQ-2 depression index in all the specifications (except for the female specification in wave 3). Furthermore, while the coefficient of childcare was larger in the male specification in either wave, an interaction of childcare and gender indicated that the difference was not statistically significant (results available on request). As expected, hunger was positively associated with worsening mental health while relative to Africans, coloureds and whites had worse mental health scores. (The official racial classifications in South Africa are Africans (indigenous black people), coloureds (mostly of mixed ancestry), Indians, and whites (people with Caucasian ancestry)). Relative to individuals who felt not at risk of contracting COVID-19, those who felt at risk had worse mental health. Also, the more educated had worse depression outcomes in wave 2 while the relationship virtually disappeared in wave 3. The statistical significance of the cut-offs indicate that the various categories should not be combined, thus lending support to our preferred ordered logit specification over a binary specification [32].

To test if family structure affected the relationship between childcare and depression, we re-estimated the above models, restricting the sample to individuals co-resident with their children. The results were similar, only generally slightly higher in magnitude to those reported in Table 2 (results available on request).

To mitigate the possibility of reverse causality between depression and childcare, we also modelled depression as a function of lagged childcare hours. The results indicate a positive and statistically significant relationship in the female specification, with the coefficient in the general specification marginally insignificant (p = 0.1)–see Table A1 in S1 Appendix.

To test whether the positive association between childcare and depression was moderated by childcare-induced labour market outcomes, we exploited a number of labour market outcomes. Respondents were asked a triad of questions: whether childcare stopped them from going to work or made work difficult; if childcare prevented them from working the same number of hours as they used to; and whether childcare prevented them from searching for work. We interacted childcare hours with each of these variables in wave 2 (these variables do not exist in wave 3). The results (Table 3) indicate that the positive relationship between hours devoted to childcare and depression was at least moderated by childcare preventing male caregivers from working or making work difficult for them. Childcare preventing job search seemed to also be a moderating factor even though it was only statistically significant in the general population.

**Table 3:** Labour market-related factors possibly mediating the relationship between depression and childcare.

	(1)	(2)	(3)	(4)	(5)	(6)	(7)	(8)	(9)
	Prevented work or made it difficult			Caused a reduction in hours of work			Prevented caregiver from searching for jobs		
VARIABLES	Female	Male	All	Female	Male	All	Female	Male	All
Childcare hours per weekday	0.016	0.007	0.014*	0.015	0.014	0.015*	0.014	0.011	0.012
	(0.010)	(0.011)	(0.008)	(0.010)	(0.012)	(0.009)	(0.010)	(0.011)	(0.008)
Childcare prevented/impeded work (kidstopwork)	0.230	-0.022	0.171						
	(0.242)	(0.331)	(0.189)						
Childcare hours per weekday* kidstopwork	0.012	0.045*	0.019						
	(0.015)	(0.024)	(0.013)						
Childcare caused reduced work hours (kidsredhrs)				0.265	0.147	0.215			
				(0.273)	(0.317)	(0.206)			
Childcare hours per weekday* kidsredhrs				0.011	0.025	0.015			
				(0.018)	(0.027)	(0.015)			
Childcare prevented job search (kidsprevsearch)							0.404*	-0.132	0.173
							(0.228)	(0.413)	(0.212)
Childcare hours per weekday* kidsprevsearch							0.016	0.040	0.026*
							(0.013)	(0.030)	(0.014)
Location (Reference = lives in formal location)									
Lives in informal location	0.230	0.103	0.170	0.219	0.090	0.166	0.204	0.083	0.157
	(0.214)	(0.246)	(0.169)	(0.212)	(0.255)	(0.166)	(0.206)	(0.240)	(0.163)
Lives in traditional location	-0.117	0.677**	0.174	-0.138	0.628**	0.149	-0.168	0.643**	0.134
	(0.195)	(0.295)	(0.169)	(0.195)	(0.292)	(0.169)	(0.198)	(0.289)	(0.169)
Lives on a farm or smallholding	-0.406*	0.207	-0.154	-0.419*	0.200	-0.163	-0.432*	0.220	-0.167
	(0.224)	(0.390)	(0.219)	(0.224)	(0.400)	(0.222)	(0.224)	(0.392)	(0.219)
Married or cohabiting	-0.041	-0.300	-0.180	-0.029	-0.255	-0.161	-0.040	-0.283	-0.173
	(0.128)	(0.213)	(0.116)	(0.130)	(0.211)	(0.116)	(0.130)	(0.208)	(0.116)
Household experienced a decrease in main source of income in past 4 weeks	0.206	0.176	0.108	0.211	0.264	0.152	0.136	0.217	0.099
	(0.184)	(0.246)	(0.163)	(0.184)	(0.248)	(0.164)	(0.180)	(0.241)	(0.159)
Someone in household experienced hunger in last 7 days due to lack of food	0.728***	0.345	0.559***	0.774***	0.380*	0.603***	0.655***	0.471**	0.566***
	(0.164)	(0.225)	(0.129)	(0.160)	(0.229)	(0.124)	(0.158)	(0.223)	(0.128)
Lives in a house/flat (otherwise, traditional/ informal/ other type of house)	-0.044	0.043	0.007	-0.015	0.027	0.023	0.006	-0.016	0.021
	(0.189)	(0.255)	(0.168)	(0.192)	(0.252)	(0.172)	(0.188)	(0.259)	(0.166)
Perception of COVID-19 risk (Reference = not at risk of contracting COVID-19)									
Self-perceived uncertain risk of COVID-19	0.124	0.346	0.228	0.145	0.388	0.244	0.167	0.347	0.234
	(0.203)	(0.292)	(0.166)	(0.201)	(0.286)	(0.160)	(0.200)	(0.285)	(0.165)
Self-perceived at risk of COVID-19	0.236	0.198	0.249*	0.236	0.225	0.252*	0.285	0.193	0.262*
	(0.181)	(0.211)	(0.134)	(0.186)	(0.208)	(0.137)	(0.184)	(0.210)	(0.137)
Years of schooling	0.022	0.026	0.022	0.021	0.020	0.020	0.017	0.025	0.019
	(0.018)	(0.031)	(0.016)	(0.018)	(0.033)	(0.017)	(0.018)	(0.032)	(0.016)
Age (years)	0.001	-0.011	-0.003	0.001	-0.014	-0.004	0.003	-0.013	-0.002
	(0.005)	(0.010)	(0.005)	(0.005)	(0.010)	(0.005)	(0.005)	(0.009)	(0.005)
Race (Reference = African)									
Coloured	1.003***	1.695***	1.277***	0.970***	1.675***	1.258***	0.903***	1.709***	1.242***
	(0.280)	(0.560)	(0.242)	(0.281)	(0.580)	(0.255)	(0.278)	(0.560)	(0.223)
Asian/Indian	0.583	0.223	0.241	0.585	0.240	0.254	0.502	0.246	0.244
	(1.067)	(0.607)	(0.357)	(1.060)	(0.630)	(0.363)	(0.888)	(0.626)	(0.342)
White	1.185***	2.022***	1.519***	1.181***	2.066***	1.541***	1.209***	2.093***	1.554***
	(0.340)	(0.504)	(0.287)	(0.340)	(0.500)	(0.285)	(0.326)	(0.497)	(0.284)
Household member(s) received grant	0.004	-0.093	-0.019	-0.001	-0.041	-0.003	-0.104	-0.078	-0.072
	(0.224)	(0.225)	(0.153)	(0.223)	(0.230)	(0.153)	(0.214)	(0.230)	(0.157)
Number of co-resident children	-0.044	0.005	-0.020	-0.039	-0.002	-0.017	-0.034	-0.000	-0.013
	(0.040)	(0.062)	(0.032)	(0.040)	(0.063)	(0.032)	(0.039)	(0.063)	(0.031)
Male			0.272*			0.263*			0.258*
			(0.149)			(0.149)			(0.148)
Cutoff 1	0.844*	0.364	0.786*	0.841*	0.315	0.796**	0.808*	0.311	0.773*
	(0.497)	(0.642)	(0.403)	(0.487)	(0.643)	(0.400)	(0.484)	(0.638)	(0.400)
Cutoff 2	1.515***	0.905	1.396***	1.506***	0.848	1.399***	1.484***	0.821	1.374***
	(0.510)	(0.643)	(0.409)	(0.501)	(0.644)	(0.407)	(0.498)	(0.638)	(0.406)
Cutoff 3	2.284***	1.655**	2.145***	2.272***	1.600**	2.147***	2.258***	1.575**	2.127***
	(0.508)	(0.666)	(0.413)	(0.498)	(0.663)	(0.410)	(0.494)	(0.661)	(0.409)
Cutoff 4	3.203***	2.702***	3.103***	3.188***	2.635***	3.101***	3.191***	2.626***	3.095***
	(0.531)	(0.655)	(0.411)	(0.520)	(0.652)	(0.408)	(0.520)	(0.647)	(0.406)
Cutoff 5	4.040***	3.602***	3.952***	4.026***	3.516***	3.946***	4.031***	3.520***	3.946***
	(0.543)	(0.708)	(0.451)	(0.534)	(0.707)	(0.449)	(0.532)	(0.696)	(0.445)
Cutoff 6	4.388***	4.089***	4.355***	4.375***	3.996***	4.348***	4.379***	4.013***	4.352***
	(0.538)	(0.762)	(0.477)	(0.527)	(0.753)	(0.474)	(0.525)	(0.744)	(0.470)
F	4.969	3.409	7.183	4.484	3.528	7.610	5.230	3.170	7.446
p	0	2.19e-06	0	1.24e-09	1.05e-06	0	0	9.58e-06	0
**Observations**	**2,080**	**1,025**	**3,105**	**2,076**	**1,023**	**3,099**	**2,091**	**1,019**	**3,110**

Note: Model is ordered logit; Outcome is PHQ-2 depression scores; Samples restricted to individuals who spent time on childcare; All statistics account for survey design and non-random attrition

*, **, *** indicate statistical significance at 10%, 5% and 1% level of significance; Standard errors in parentheses.

## Discussion

COVID-19 has caused significant disruptions, not least in the area of childcare. The pandemic brought about movement restrictions and school closures, resulting in families having to shoulder additional childcare responsibilities. Even with the progressive relaxation of lockdowns and the gradual re-opening of schools, many schools adopted remote teaching and learning, with the implication that parents and caregivers had to spend more time than usual helping children with schoolwork. Furthermore, many parents still felt anxious about taking their children to school partly due to the fear of them contracting the virus and/or infecting family members. For instance, about 72% of adults in the second wave of the NIDS-CRAM survey indicated that they were very worried about children returning to school [33]. Thus, school closure regulations, concerns for children’s safety and economic hardship caused by the pandemic resulted in parents and guardians shouldering more childcare responsibilities than normal. Sometimes, such additional childcare responsibilities occurred in the context of parents and caregivers carrying on with their usual work routine as well as managing the stressful environment occasioned by the pandemic. Given the foregoing, this paper ascertained whether time spent looking after children during weekdays was associated with depression during the pandemic.

Our finding of a positive relationship between childcare and depressive symptoms concurs with other evidence uncovered especially during the pandemic. For instance, a study in Poland found that parents expressed anxiety about their children’s future while not feeling generally confident about their competence and the home-schooling solutions they adopted [16]. An Australian study highlighted the general frustration parents experienced in home-schooling or helping their children with remote learning during the pandemic. The study, which analysed Twitter comments with regard to the lockdown, revealed the physical and mental health challenges of home-schooling and its potential to negatively affect family relationships. Some of the respondents’ comments included, “… I talk to parents everyday. I’m bloody frustrated and exhausted and angry too”, and “I honestly could not do home-schooling for a term. My son would suffer academically and our relationship would suffer” [12].

Numerically, we found a stronger relationship between childcare and depressive symptoms in men than women in both waves, even though such gender differences were not statistically significant. We also note that our finding of a closing of the gender gap in childcare time between wave 2 and wave 3 contradicts earlier evidence on the change between wave 1 and wave 2 for men and women living with children as reported by Casale and Shepherd [13]. That said, the higher coefficient of time spent on childcare in the male regressions may not be unconnected with the fact that cultural norms have historically viewed childcare as largely a woman’s job [34–36]. Thus, when men are forced by circumstances like COVID-19 to spend time on childcare above the norm, such may result in elevated risk of depressive symptoms.

International evidence on the relationship between sharing of childcare/housework and mental health or subjective wellbeing is mixed [see 36 for a synthesis]. Some scholars have found evidence that spending more time on childcare is positively associated with depression or lower levels of life satisfaction [37–42]. A study of parents’ subjective wellbeing regarding time spent with their children found that mothers reported less happiness, greater stress and more fatigue in the time they spent with their children compared to the fathers’ experiences [43]. Similarly, Roeters and Gracia [44] noted that mothers found childcare time to be more stressful than fathers in the US, with fathers finding such time more meaningful. However, the relationship was nuanced, with mothers cherishing time spent with minors while finding time with adolescent children stressful while fathers found time spent with minors stressful while time spent with middle school age children was highly meaningful to them. On the other hand, Glass and Fujimoto [38] found that men who share housework responsibilities report less wellbeing than their counterparts who abide by more traditional household division of labour. However, some studies found no relationship between men’s housework responsibilities and their psychological health [39].

Regarding the South African literature, Posel and Casale [45] found that the presence of children in the household was associated with lower subjective wellbeing for women while such a relationship did not exist for men. While our outcome and childcare indicator are not identical to theirs, it is clear that our findings do not necessarily concur with their study given that both men and women experienced a positive relationship between childcare and worse depression outcome.

Hunger was significantly correlated with depression. This is not unexpected given the demonstrated evidence of a relationship between food insecurity and mental health [46]. Our finding of worse depressive health outcomes among non-Africans during the COVID-19 pandemic in South Africa concurs with earlier evidence in this regard [19].

We also found that perceiving oneself to be at risk of contracting COVID-19 was significantly associated with worse mental health. This finding echoes an earlier study which indicated that the fear of COVID-19 was significantly associated with depression and anxiety in the US [47]. However, we found that being uncertain about one’s risk of contracting the disease had mixed results. Furthermore, we view as worrisome our finding of an increase in the proportion of the population who perceived themselves to not be at risk of contracting COVID-19 as well as a decline in the proportion who viewed themselves at risk of contracting the virus. This is especially concerning as the country has entered a second wave of COVID-19. It is even more concerning given that South Africa has reported a more infectious and perhaps more deadly strain of the virus [48].

As earlier indicated, the labour market played an important role in moderating the relationship between childcare and depression. We found that childcare preventing or impeding caregivers from working played a significant role in its association with depression especially among males, while the moderating role of childcare preventing job search was only statistically significant in the population. However, it did not appear that childcare reducing number of hours worked played a significant role in moderating the relationship between childcare and depression. While a number of studies have found that parents (especially mothers) quit the workforce during the child-rearing phase, much of the focus has been on the career effects of such work interruptions [see e.g. 49]. An exception is a study in Canada which found that a childcare subsidy policy which increased female labour force participation resulted in a worsening of life satisfaction for higher educated women but an improvement in life satisfaction among less educated women [50]. Studies examining the mental health consequences of childcare for both men and women in the context of a pandemic are virtually non-existent to our best knowledge. Our finding that childcare-related work cessation/impediment or inability to search for work intensified the relationship between childcare and depression is indeed novel especially in South Africa.

Our finding that women spent more time on childcare relative to men is not surprising and has been found elsewhere. For instance, Del Boca, Oggero [51] found that in Italy, most of the extra housework and childcare associated with COVID-19 were borne by women. However, childcare activities were more equally shared among couples than other housework activities. In a multi-country study involving academics from France, Germany, Turkey, Norway, Sweden, Italy, the UK and the USA, having children disproportionately affected the amount of housework done by female academics compared to their male colleagues, suggesting that women were more likely to engage in childcare than men in similar occupations [52]. Moreover, the narrowing of the “childcare burden gap” between women and men as found in this study conforms to earlier assertions made about the pandemic, where it was posited that the pandemic would result in fathers assuming greater primary responsibility for childcare, thereby eroding social norms which disfavour women in terms of housework and childcare [53].

A limitation of the study is that the aforementioned relationship between childcare and mental health is not causal. Indeed, there is evidence that poor mental health can affect childcare [12]. However, we suspect that such reverse causality issues would at most attenuate the observed relationship given that pre-existing poor mental health would likely reduce the amount of time parents spend with their children [12]. To the extent that this is true, our estimates may be viewed as lower bounds of the impact of childcare on mental health in South Africa during the COVID-19 pandemic. Furthermore, childcare was self-reported. While this does not mean that it is biased, it would have been desirable to have a more objective analogue obtained via, say, the diary method if only for sensitivity and triangulation purposes. Finally, we think that the national nature of this analysis does not lend its conclusions for applicability to the wider Southern Africa region. However, it is important to understand how these relationships play out in the region as the various governments grapple with pandemic response. With the availability of relevant data, we believe that it will be worth ascertaining the nature of the above relationships in these other contexts especially for the purpose of validation. This, therefore, forms an important agenda for future research.

## Conclusions

This paper has analysed the relationship between childcare and depression in South Africa during the COVID-19 pandemic across gender groups. First, we find evidence of substantial need for childcare services especially given the economic devastation caused by the pandemic as well as concerns over the safety of formal childcare services. These issues have resulted in a substantial childcare burden for parents and guardians, thus raising the possibility of adverse mental health outcomes. We find that though there were no significant gender differences in probable depression in the July-August and November-December 2020 periods, mental health outcomes worsened in the population over both periods. Women spent significantly more time looking after children than men. However, the gender gap in the average time spent on childcare during weekdays declined. The results indicate that spending more time looking after children is associated with worse depression outcome, with the relationship stronger among men than women in numerical terms especially in the November-December 2020 period. Childcare preventing/hindering (especially male) caregivers from working, and childcare preventing job seekers from job hunting moderate the relationship between childcare and depression. This study posits that policy response to the pandemic and pandemic control measures must prioritize the mental health of parents and guardians especially with the emergence of a second wave of the pandemic in South Africa. Perhaps, measures like encouraging employers to implement flexible work schedules, encouraging greater communication between parents and school authorities in the event of further school closures and job search assistance to parents and guardians may be helpful in ameliorating the mental health effects of childcare responsibilities during the pandemic.

## Supporting information

S1 Appendix Table A1. Relationship between depression and lagged childcare.
(DOCX)
