# Supplementary material for: Childcare and depression during the coronavirus pandemic in South Africa: A gendered analysis
Source: PLoS One. 2021 Aug 6;16(8):e0255183. doi: 10.1371/journal.pone.0255183 (PMC8345885; doi:10.1371/journal.pone.0255183)
Supplement: S1 Appendix — Table A1. Relationship between depression and lagged childcare. (DOCX) [file pone.0255183.s001.docx]

# **S1 Appendix.**

Table A1. Relationship between depression and lagged childcare.

|  | (1) | (2) | (3) |
| --- | --- | --- | --- |
|  | Female | Male | All |
|  |  |  |  |
| Lagged Childcare hours per weekday | 0.010** | -0.002 | 0.006 |
|  | (0.005) | (0.007) | (0.004) |
| **Location (Reference = lives in formal location)** |  |  |  |
| Lives in informal location | 0.279** | 0.117 | 0.230** |
|  | (0.115) | (0.143) | (0.090) |
| Lives in traditional location | -0.070 | -0.077 | -0.067 |
|  | (0.127) | (0.163) | (0.100) |
| Lives on a farm or smallholding | -0.179 | -0.031 | -0.128 |
|  | (0.148) | (0.204) | (0.119) |
| Married or cohabiting | -0.137 | -0.050 | -0.113* |
|  | (0.084) | (0.123) | (0.068) |
| Not employed | -0.164* | -0.134 | -0.157** |
|  | (0.085) | (0.113) | (0.067) |
| Household experienced a decrease in main source of income in past 4 weeks | 0.141* | 0.280** | 0.190*** |
|  | (0.085) | (0.117) | (0.069) |
| Someone in household experienced hunger in last 7 days due to lack of food | 0.766*** | 0.873*** | 0.793*** |
|  | (0.101) | (0.139) | (0.081) |
| Lives in a house/flat (otherwise, traditional/ informal/ other type of house) | 0.053 | -0.072 | 0.008 |
|  | (0.100) | (0.133) | (0.079) |
| **Perception of COVID-19 risk (Reference = not at risk of contracting COVID-19)** |  |  |  |
| Self-perceived uncertain risk of COVID-19 | 0.133 | -0.014 | 0.073 |
|  | (0.144) | (0.191) | (0.115) |
| Self-perceived at risk of COVID-19 | 0.512*** | 0.313*** | 0.439*** |
|  | (0.087) | (0.113) | (0.069) |
| Years of schooling | 0.028** | -0.013 | 0.014 |
|  | (0.013) | (0.017) | (0.010) |
| Age (years) | 0.002 | -0.004 | -0.000 |
|  | (0.003) | (0.005) | (0.003) |
| **Race (Reference = African)** |  |  |  |
| Coloured | 0.865*** | 1.137*** | 0.974*** |
|  | (0.147) | (0.184) | (0.114) |
| Asian/Indian | 0.611 | 0.671 | 0.625* |
|  | (0.571) | (0.463) | (0.354) |
| White | 0.716*** | 1.036*** | 0.861*** |
|  | (0.230) | (0.259) | (0.171) |
| Male (otherwise, female) |  |  | 0.110 |
|  |  |  | (0.070) |
| Household member(s) received grant | 0.028 | 0.075 | 0.068 |
|  | (0.126) | (0.125) | (0.088) |
| Number of co-resident children | 0.027 | 0.032 | 0.029 |
|  | (0.022) | (0.029) | (0.017) |
| Cutoff 1 | 0.708** | -0.129 | 0.468** |
|  | (0.298) | (0.351) | (0.231) |
| Cutoff 2 | 1.392*** | 0.502 | 1.130*** |
|  | (0.299) | (0.352) | (0.232) |
| Cutoff 3 | 2.124*** | 1.106*** | 1.811*** |
|  | (0.301) | (0.353) | (0.233) |
| Cutoff 4 | 3.089*** | 2.143*** | 2.800*** |
|  | (0.306) | (0.358) | (0.237) |
| Cutoff 5 | 3.929*** | 2.915*** | 3.610*** |
|  | (0.313) | (0.366) | (0.242) |
| Cutoff 6 | 4.328*** | 3.222*** | 3.969*** |
|  | (0.319) | (0.371) | (0.246) |
|  |  |  |  |
| Pseudo R2 | 0.025 | 0.026 | 0.024 |
| p | 0 | 0 | 0 |
| Observations | 2,119 | 1,272 | 3,391 |

Note: Model is ordered logit; Outcome is PHQ-2 depression scores; *, **, *** indicate statistical significance at 10%, 5% and 1% level of significance; Standard errors in parentheses.
